# The genome sequence of celery (*Apium graveolens* L.), an important leaf vegetable crop rich in apigenin in the Apiaceae family

**DOI:** 10.1038/s41438-019-0235-2

**Published:** 2020-01-06

**Authors:** Meng-Yao Li, Kai Feng, Xi-Lin Hou, Qian Jiang, Zhi-Sheng Xu, Guang-Long Wang, Jie-Xia Liu, Feng Wang, Ai-Sheng Xiong

**Affiliations:** State Key Laboratory of Crop Genetics and Germplasm Enhancement, Ministry of Agriculture and Rural Affairs Key Laboratory of Biology and Germplasm Enhancement of Horticultural Crops in East China, College of Horticulture, Nanjing Agricultural University, 1 Weigang, Nanjing, 210095 China

**Keywords:** DNA sequencing, Genome evolution

## Abstract

Celery (*Apium graveolens* L.) is a vegetable crop in the Apiaceae family that is widely cultivated and consumed because it contains necessary nutrients and multiple biologically active ingredients, such as apigenin and terpenoids. Here, we report the genome sequence of celery based on the use of HiSeq 2000 sequencing technology to obtain 600.8 Gb of data, achieving ~189-fold genome coverage, from 68 sequencing libraries with different insert sizes ranging from 180 bp to 10 kb in length. The assembled genome has a total sequence length of 2.21 Gb and consists of 34,277 predicted genes. Repetitive DNA sequences represent 68.88% of the genome sequences, and LTR retrotransposons are the main components of the repetitive sequences. Evolutionary analysis showed that a recent whole-genome duplication event may have occurred in celery, which could have contributed to its large genome size. The genome sequence of celery allowed us to identify agronomically important genes involved in disease resistance, flavonoid biosynthesis, terpenoid metabolism, and other important cellular processes. The comparative analysis of apigenin biosynthesis genes among species might explain the high apigenin content of celery. The whole-genome sequences of celery have been deposited at CeleryDB (http://apiaceae.njau.edu.cn/celerydb). The availability of the celery genome data advances our knowledge of the genetic evolution of celery and will contribute to further biological research and breeding in celery as well as other Apiaceae plants.

## Introduction

**Fig. 1:**
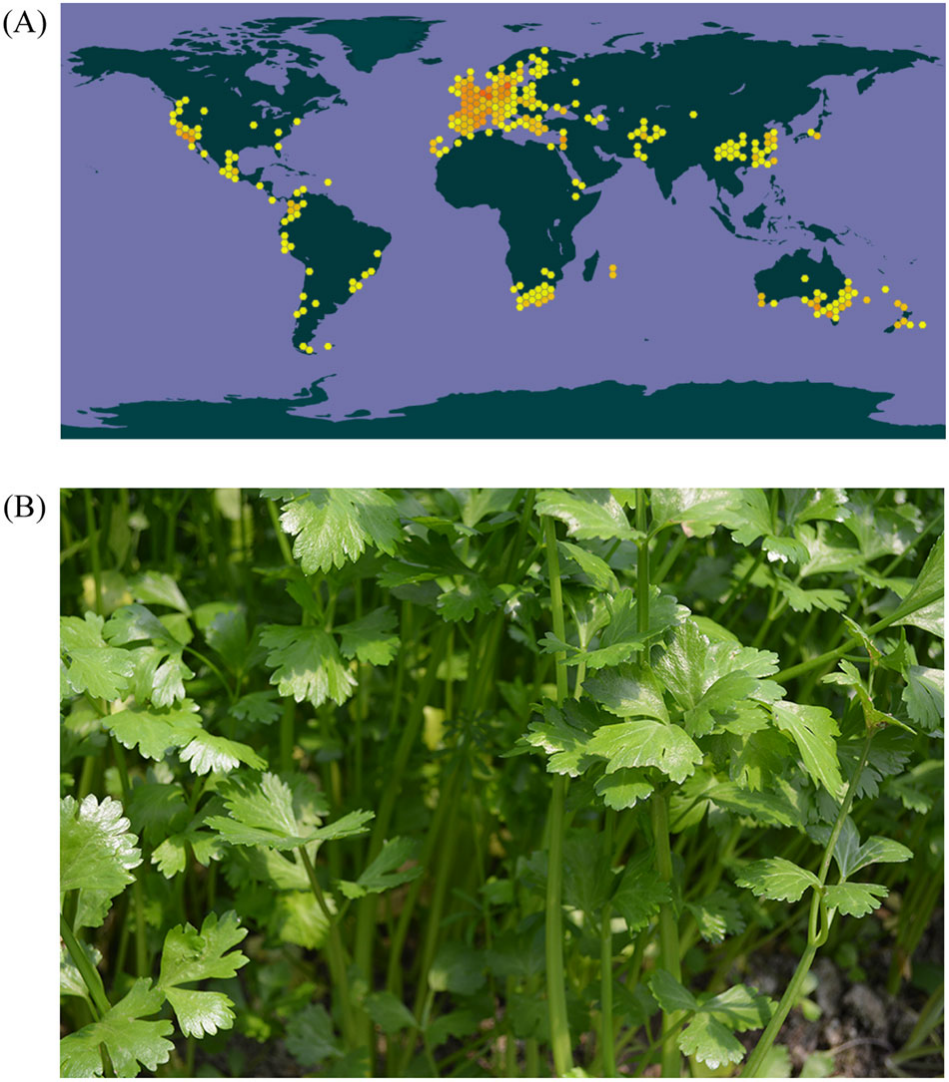
Georeferenced records and an image of celery. **a** Georeferenced records of celery on the world map. **b** Image of celery. The data set was obtained from the GBIF website (www.gbif.org). The colored hexagons represent the locations of georeferenced celery records.

Celery (*Apium graveolens* L.) is an annual or biennial herbaceous plant in the Apiaceae family that originated in the Mediterranean and the Middle East. It is a popular vegetable crop and is widely cultivated in Europe, East Asia, southeastern Oceania, and southern Africa (Fig. 1a). The whole celery plant exhibits aromatic flavor, and its leaf blades and petioles are the main edible organs (Fig. 1b). In addition to containing common nutrients such as vitamins, proteins and carbohydrates, celery contains flavonoids, carotenoids, terpenoids, and unsaturated fatty acids that exhibit biological activity and physiological functions in human beings^1–4^.

Flavonoids are a class of natural products that are widely found in plants, most of which exist in the form of glycosides. Vegetables and fruits are the predominant dietary sources of flavonoids. Flavonoids are one of the most important types of secondary metabolites in celery, mainly comprising apigenin, kaempferol, quercetin, and luteoli^5^. In particular, the content of apigenin in celery is higher than that in other plants^6^. Many studies on the isolation, identification, and application of flavonoids in celery have been carried out^4,7,8^. Apigenin and luteolin exhibit a wide range of pharmacological effects, including anti-bacterial, anti-oxidation, and cardiovascular protective effects^9,10^. Many terpenoids and aromatic compounds are present in celery tissues, which contribute to its unique fragrance. Some studies have shown that terpenoids from celery seed oil exert strong lethal effects on *Aedes aegypti*^11^. Effective celery components such as allergenic proteins, apigenin, and anthocyanin have caused wide public concern^12–15^. However, the regulatory mechanisms of the effective components of celery remain unclear.

Whole-genome sequencing has overcome the limitations of traditional basic research and generated massive data that greatly promote research on plants. The genome sequence of *Arabidopsis thaliana* was completed and published in 2000, providing the first reported sequencing data for higher plants^16^. Since then, the genomes of many species have been sequenced and published^17–21^. Apiaceae is one of the largest families of flowering plants, with ~4000 species classified into 434 genera. The Apiaceae family contains several vegetable and spice crop species. However, the lack of complete genome data places greatly constrains the improvement of Apiaceae crops. The molecular resources for Apiaceae species are underdeveloped. Thus far, a reported carrot genome represents the only genome data for the Apiaceae family^20,22^.

In this paper, we report the genome sequence of celery, which is one of the most economically important species of the Apiaceae family. The ~189× coverage sequence of the celery genome provides information on the overall organization, gene content, and structural components of the DNA. Transcription factors, disease resistance genes, apigenin and terpenoid biosynthesis-related genes, and other functional genes were also identified in this study. All of the data not only provide valuable resources for basic and applied research on celery but also lay the foundation for the analysis of the evolution and comparative genomics of celery and Apiaceae species.

## Materials and methods

### Plant material, DNA preparation, and genome sequencing

The celery material used in this study was the highly inbred line Q2-JN11, which was derived via the forced selfing of “Jinnan Shiqin”. The seeds of Q2-JN11 were collected in the State Key Laboratory of Crop Genetics and Germplasm Enhancement, Nanjing Agricultural University, China. High-quality genomic DNA was extracted from the young leaves of celery for genome sequencing using a modified CTAB method^23^.

The genome was sequenced using the HiSeq 2000 platform. Libraries with six different insert sizes (180 bp, 500 bp, 800 bp, 2 kb, 5 kb, and 10 kb) were prepared from Q2-JN11 DNA and then sequenced according to standard Illumina protocols at the Beijing Genomics Institute-Shenzhen (BGI-Shenzhen).

### Genome assembly

The raw reads generated from each library were preprocessed to facilitate their assembly. Low-quality reads were filtered out, and reads with adapter contamination were removed using CutAdapt^24^. The paired-end reads were assembled into contigs using SOAPdenovo2 (http://soap.genomics.org.cn/soapdenovo.html)^25^ with the multi-kmer option (*k*-mer = 63). We then aligned all usable paired-end reads onto the contig sequences and used mate-pair information on the order of the estimated insert size (180 bp to 10 kb) to construct scaffolds. Gaps within the scaffolds were closed using GapCloser. BUSCO v3 was employed to measure the genome assembly using the Eudicotyledons (odb10) database with default parameters^26^.

### Gene prediction and annotation

The presence of possible celery-specific transcripts within a region was analyzed using Augustus 3.2.2 and SNAP^27,28^. For similarity-based gene prediction, five sequenced plants (*A. thaliana*, *Mimulus guttatus*, *Solanum tuberosum*, *Solanum lycopersicum*, and *Daucus carota*) were selected, and the protein sequences of these species were downloaded from Phytozome v12 (http://www.phytozome.net). BLAST (identity ≥ 0.95; coverage ≥ 0.90) was used to identify ESTs, mRNAs, and proteins with significant similarity to the celery genome sequence. The identity and coverage thresholds used in the alignment were determined according to the methods employed for the carrot and potato genomes^20,29^. Manual curation and several rounds of refinement using Exonerate v.2.2.0 were performed to realign or polish the sequences following filtering and clustering^30^. The functional annotation of protein-coding genes was achieved using BLASTP (*E* value of 1 × 10^−4^) against the NCBI non-redundant protein sequence (nr), TrEMBL, Swiss-Prot, Gene Ontology (GO), and Kyoto Encyclopedia of Genes and Genomes (KEGG) databases^31,32^. Blast2GO^33^ was used to obtain the relevant GO ID, and WEGO^34^ was applied to illustrate the distribution of gene classifications.

### Noncoding RNAs

Noncoding RNAs were identified by searching against various RNA libraries. tRNA scan-SE (version 1.3.1) was employed to search for reliable tRNA positions^35^. Small nuclear RNAs (snRNAs) and microRNAs (miRNAs) were searched via a two-step method involving initial alignment with BLAST followed by searching with INFERNAL^36^ against the Rfam database (v12.0)^37^.

### Repetitive elements

Transposable elements in the celery assembly were identified using a combination of homology-based and *de novo* approaches. The known repetitive elements were identified with RepeatMasker (version 3.3.0) against the Repbase library^38^. RepeatModeler (http://www.repeatmasker.org/RepeatModeler.html) was then used after masking the known repetitive elements. The consensus sequences generated with RepeatModeler were searched against the SWISS-PROT database using BLASTX, and consensus sequences with significant similarity to protein-coding genes were eliminated. Finally, RepeatMasker was run on the genome sequences using the consensus sequence as a library.

### Gene family analysis

We used OrthoMCL to define a gene family as a group of genes descending from a single gene in the last common ancestor of the considered species^39^. The protein-coding genes of *M. guttatus* v2.0, *S. lycopersicum* iTAG2.4, *D. carota* v2.0, and *A. thaliana* TAIR10 were downloaded from Phytozome v12 (http://www.phytozome.net)^40^. The longest protein sequence prediction was employed to perform all-against-all comparisons using BLASTP. All protein sequences were compared against a database containing protein data sets for all the species under an *E* value of 1 × 10^−5^. The BLASTP results were further filtered if the aligned region length was <50% of any of the aligned two protein sequences. The Markov cluster (MCL) algorithm was then used to cluster the BLASTP results into groups of homologous proteins with an inflation parameter of 1.5. To analyze the evolution of gene families, CAFE was used to calculate the number of expansions and contractions of gene families^41^.

### Phylogenetic analysis

To build the phylogenetic tree, redundant sequences (90% identity or more) from the same organism were removed using CD-HIT^42^. Then, homolog clusters were predicted by comparing each pair of the nine plant genomes (*A. graveolens*, *D. carota*, *M. guttatus*, *S. tuberosum*, *S. lycopersicum*, *Oryza sativa*, *Coffea canephora, Actinidia chinensis*, and *A. thaliana*) using OrthoMCL. The protein sequences for clusters containing a single-copy gene of each species were aligned with MUSCLE v3.8.31^43^. Then, positions showing poor alignment were eliminated with G blocks (version 0.91b). All alignments were subsequently concatenated to one super alignment. The phylogeny was reconstructed by the neighbor-joining method using the Jones-Taylor-Thornton model^44^. The reliability of the tree topology was measured by bootstrapping (1000 replications). Evolutionary analysis was conducted in MEGA7^45^. The species divergence time in the phylogenetic tree was estimated via the Bayesian relaxed molecular clock (BRMC) method using the program MULTIDIVTIME, which was implemented in the Thornian Time Traveller (T3) package^46,47^. To estimate the synonymous substitutions per synonymous site (*Ks*), all paralogous gene pairs were analyzed with the maximum likelihood method in the PAML program^48^.

### Transcription factors

Reference information was collected from PlnTFDB^49^, an integrated plant transcription factor database including genes from *A. thaliana*, *Populus trichocarpa*, and *Oryza sativa* (http://plntfdb.bio.uni-potsdam.de). For each transcription factor family, conserved domains were used as queries for searching similar sequences in the celery genome. The protein domains of the identified transcription factors were classified using the Pfam database.

### Functional genes

Celery resistance-related genes were identified based on the most conserved motif structures of plant resistance proteins, including coiled coil (CC), Toll/Interleukin-1 receptor (TIR), nucleotide-binding site (NBS), and leucine-rich repeat (LRR) finger domains. Conserved motifs were derived from domain profiles retrieved from the PFAM, PANTHER, PRINTS, PROSITE, SMART, and SUPERFAMILY databases. PAIRCOIL2 was used to specifically detect CC domains.

To identify the genes involved in the flavonoid biosynthesis pathway in celery, the assembled genes were annotated with the corresponding Enzyme Commission numbers against the KEGG database. All candidate genes were further submitted to the NCBI database to obtain gene function information. Terpenoid synthase (TPS) proteins were identified by screening the celery genome sequences using HMMER3.0 software with domain models PF03936 and PF01397 as queries^50^.

### Data access

The whole-genome sequences of celery have been deposited at CeleryDB under accession version 1.0^51^. The genome data can be accessed at http://apiaceae.njau.edu.cn/celerydb.

## Results

### Genome sequencing and assembly

For celery genome sequencing, a total of 68 genomic libraries with three small insert sizes (180 bp, 500 bp, and 800 bp) and three large insert sizes (2 kb, 5 kb, and 10 kb) were prepared. We generated raw sequences from the next-generation sequencing platform HiSeq 2000. After filtering, a total of 600.8 Gb of clean data were obtained from the paired-end libraries with different insert sizes (Supplementary Table S1). The genome size was estimated to be 3.18 Gb based on the 17-mer depth distribution (Supplementary Fig. S1 and Table S2). All clean data were assembled into contigs and scaffolds using SOAP de novo, resulting in a final assembly of 2.21 Gb with N50 sizes of 13,108 bp for contigs and 35,567 bp for scaffolds (Tables 1 and 2). The total clean data generated represented 188.93× coverage of the estimated celery genome, and our assembly accounted for ~70% of the estimated genome. The assembled genome exhibited highly complete Benchmarking Universal Single-Copy Orthologs (BUSCO) (90.8%) (Supplementary Table S3). Compared with another Apiaceae species, carrot, the genome size of celery is much greater. The percentage of the GC content in celery genome was 35.35%, which was close to those in the genomes of carrot (34.80%), *Arabidopsis* (36.06%), and tomato (34.05%) but lower than those in tea tree (42.31%) and rice (43.57%) (Supplementary Table S4).

**Table 1:** Statistics of the celery genome assembly.

Feature	Value
Genome size	2.21 Gb
Genome GC%	35.35%
Gene number	34,277
Gene no. per 100 kb	1.44
Average gene length (bp)	3267
Exon region GC (%)	42.06%
Exon number	180,591
Average exon length (bp)	243.48
Exon no. per gene	5.27

**Table 2:** Statistics of the assembly size of the contigs and scaffolds of celery.

Property	Contig	Scaffold
Min sequence length (bp)	500	500
Max sequence length (bp)	228,328	556,749
Total sequence number	432,762	257,842
N50 length (bp)	13,108	35,567
N90 length (bp)	1136	4841
*N* number	648,982	280,637, 212
*N* rate (%)	0.031	11.8
Total sequence length (bp)	2,017,581,028	2,372,941,895

### Repetitive sequence analysis

*De novo* repeat identification using RepeatMasker and homology analysis against the RepBase library showed that repetitive DNA (excluding low-complexity sequences) accounted for 68.88% of the genome. The classification of the observed transposable elements into known classes revealed that the majority of repetitive sequences were LTR retrotransposons (44.07%), whereas 3.30% and 2.76% of the repeat element types were DNA transposons and simple repeat elements, respectively (Table 3).

**Table 3:** Repeat element analysis in the celery genome.

Repeat elements	Copies (numbers)	Repeat size (bp)	Percentage of the assembled genome (%)
DNA transposon	157,948	78,298,900	3.30
LINE	42,679	34,853,999	1.47
Low complexity	174,767	9,739,053	0.41
LTR/Copia	573,134	481,821,466	20.30
LTR/Gypsy	605,914	519,802,870	21.91
LTR/others	58,772	44,248,275	1.86
SINE	4272	454,864	0.02
Satellite	7993	8,983,101	0.38
Simple repeat	860,188	65,493,490	2.76
Unknown	453,513	390,743,993	16.47
Total	2,939,180	1,634,440,011	68.88

The fraction of repetitive sequences in the celery genome (68.88%) was higher than that in the carrot (45.95%) and physic nut genomes (49.81%) but lower than that detected in the genomes of tea tree (80.89%) and ginkgo (76.58%) (Supplementary Table S5). In comparison, celery, tea tree, and ginkgo exhibit larger genome sizes than carrot and physic nut. In addition, LTRs occupied the absolute dominant position among the repetitive sequences of these five plants. In this study, the activity of LTRs at the molecular level was analyzed. The amplification of celery LTR elements was relatively active ~2.5~ 4 Mya (million years ago) and 5 Mya, which was close to the recent whole-genome duplication (WGD) event that occurred in celery 1.9 Mya (Supplementary Fig. S2). Previous studies on sequenced plant genomes have shown that the proportion of repetitive sequences is one of most important factors affecting genome size^52–54^. The recently inserted LTRs may be an important factor affecting the size of the celery genome.

### Gene prediction and annotation

To predict protein-coding genes in the celery genome, *de novo* gene prediction programs and homology-based methods were combined to assemble the results. We predicted 34,277 genes with an average length of 3267 bp and a mean of 5.27 exons per gene in the celery genome (Table 1 and Supplementary S4). Compared with other species, the celery gene number was similar to that of tomato (34,727), larger than those of *Arabidopsis* (27,416) and carrot (32,113), and lower than those of tea tree (36,951) and rice (57,939) (Supplementary Table S4). In addition to protein-coding genes, we identified 891 tRNA, 116 miRNA, 374 rRNA, and 8878 snRNA genes in the celery genome, which constituted ~0.021% of the genome sequences (Table 4).

**Table 4:** Number of noncoding RNAs in the celery genome.

Type	Number	Size (bp)	Average length (bp)	Percentage of genome (%)
miRNA	116	14,759	127.23	0.00062
tRNA	891	67,306	75.54	0.00284
rRNA	374	321,240	858.93	0.01354
snRNA	8,878	938,847	105.75	0.00396

A sequence similarity search was performed against public databases to investigate putative functions. A total of 34,143 genes were annotated using the Nr, InterPro, GO, and KEGG databases (Supplementary Table S6). Based on the GO database, 16,920 genes were annotated to three gene ontology classes: biological process, cellular component, and molecular function, with 1223 functional terms (Fig. S3). The most-frequent functional clusters in the celery genome were protein binding, ATP binding and oxidation-reduction processes. By mapping to the KEGG database, 9463 genes were assigned to KEGG metabolic pathways.

**Fig. 2:**
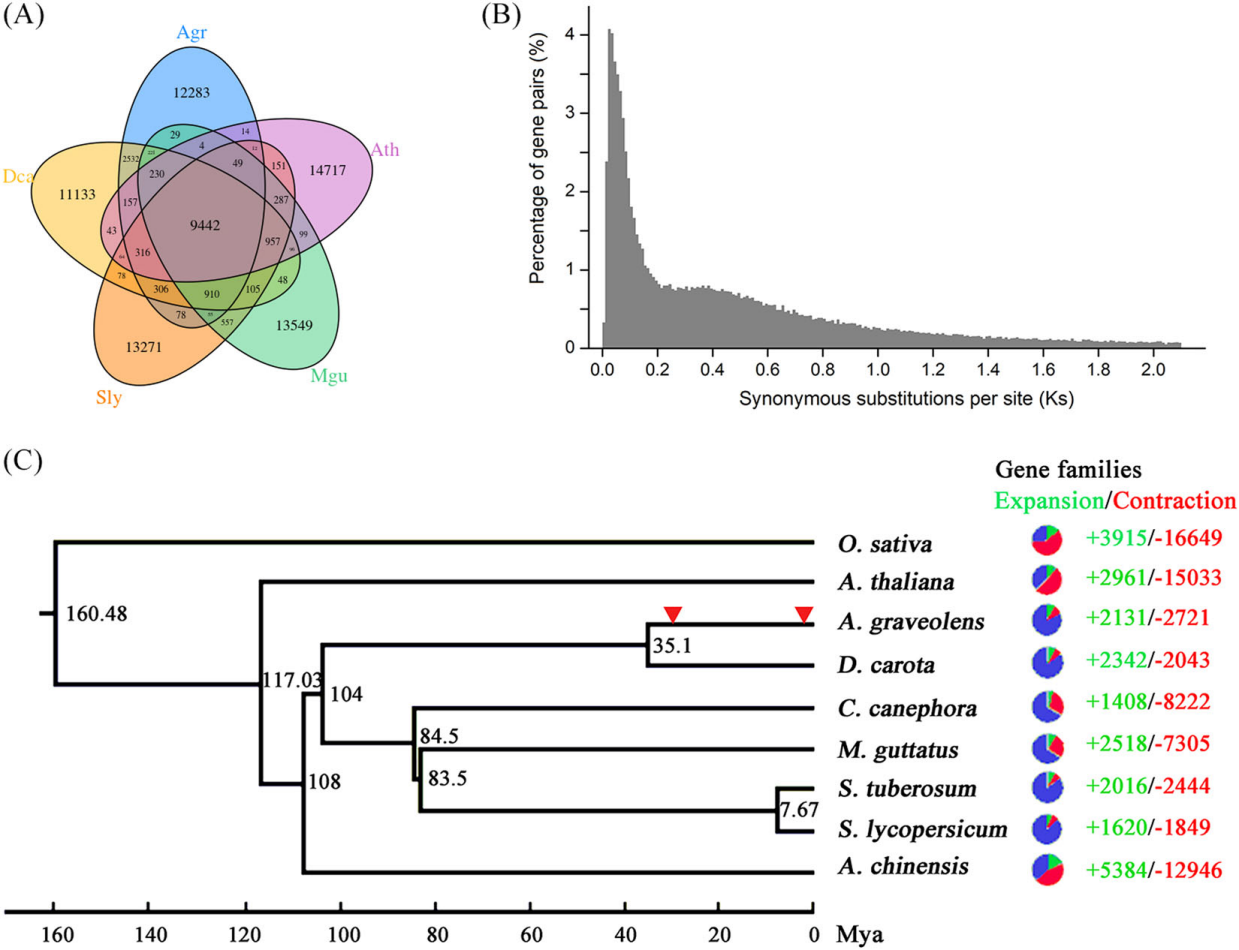
Comparative genomic and phylogenetic relationship analyses. **a** Venn diagram showing the cluster distribution of shared gene families among *A. graveolens* (Agr), *M. guttatus* (Mgu), *S. lycopersicum* (Sly), *D. carota* (Dca), and *A. thaliana* (Ath). **b** *Ks* distribution of paralogous gene pairs in the celery genome. The probability density of *Ks* was estimated using the “density” function in the R language. **c** Evolutionary analysis of celery and eight other plant species. The divergence time was estimated with the calibration time for *S. tubersum* and *S. lycopersicum*. The pie charts show the proportions of expanded (green), contracted (red), and unchanged (blue) gene families. The potential WGD events of celery are indicated with red triangles.

Based on the pair-wise protein sequence similarities, we carried out a gene family analysis of celery genes. A total of 27,549 genes were clustered in 15,164 gene families with an average size of 1.82, and 6728 of these genes did not exhibit homologous sequences (Supplementary Table S7). The number of members in the gene families varied greatly, and group 4 exhibited the largest number, including 201 genes (Supplementary Fig. S4). The gene ontology category analysis showed that the gene families that contained the most members were widely involved in biological processes such as cell recognition, serine-type endopeptidase activity, flavin adenine dinucleotide binding, protein phosphorylation, and zinc ion binding (Supplementary Table S8). For the analysis of the specific and shared gene families among species, conserved putative genes from five plants were used to identify gene family clusters. Among the total 81,793 clusters, 9442 clusters were observed in all investigated species, and 12,283 appeared to be lineage specific to celery, whereas 13,934 were shared with *D. carota*, 10,224 with *A. thaliana*, 11,168 with *S. lycopersicum*, and 10,940 with *M. guttatus* (Fig. 2a). The celery-specific genes were analyzed on the basis of the results of gene prediction and annotation (Supplementary Table S9). A total of 783 celery-specific genes corresponded to 1456 annotation results, which were divided into 155 GO terms.

### Celery-specific transcription factors and disease resistance-related genes

Within the celery genome, the percentage of transcription factors (4.95%) was slightly greater than that in the rice genome (4.80%) but lower than those in carrot (8.41%), *Arabidopsis* (7.00%), and tomato (7.09%). In the celery genome, there are six transcription factor families (FAR1, ERF, MYB, MYB-related, bHLH, and NAC) that contain >100 members. A total of 184 celery-specific transcription factors were identified from the 1698 putative transcription factors of celery, which were classified into 12 transcription factor families (Fig. S5). Similar to the percentage of total transcription factors in the celery genome, the FAR1 (59%) family yielded the largest number of celery-specific transcription factors. NAC was the second largest family of celery-specific transcription factors, with 28 members. The number of celery-specific transcription factors in both the MYB family and the MYB-related family was 10.

Celery encounters various forms of environmental stress, including a wide range of pests and pathogens that negatively affect celery growth and yield. A variety of disease resistance-related genes are induced to respond to stresses and to increase celery tolerance. NBS and carboxy-terminal LRR domains are found in the majority of R proteins^55,56^. A total of 201 NBS-containing resistance genes were identified based on resistance domain analyses in the celery genome, all of which were further classified into six groups: TIR-NBS-LRR, CC-NBS-LRR, CC-NBS, TIR-NBS, NBS-LRR, and NBS (Table 5). The total number of NBS-LRR genes was 146 in carrot^20^, 176 in *Arabidopsis*^57^, 107 in maize^58^, and 472 in rice^59^. Among these genes in the celery genome, one class, NBS, presented a markedly greater number in celery (126 genes) than in other plants including carrot (19), *Arabidopsis* (1), maize (7) and rice (25). The NBS-LRR genes present in monocotyledons and dicotyledons showed different characteristics. The CNL and NL groups accounted for a large proportion of the genes in monocotyledonous plants (maize and rice), whereas no genes encoding a TIR domain were found. However, all of the groups existed in dicotyledonous plants, but their distributions were different among species.

**Table 5:** Comparison of the numbers and classifications of genes encoding an NBS domain in celery, carrot, *Arabidopsis*, maize, and rice.

Protein domains	Letter code	Celery	Carrot	*Arabidopsis*	Maize	Rice
CC-NBS-LRR	CNL	6	57	51	58	160
TIR-NBS-LRR	TNL	0	4	92	0	0
CC-NBS	CN	47	0	5	11	7
TIR-NBS	TN	19	0	21	0	0
NBS-LRR	NL	3	66	6	31	280
NBS	N	126	19	1	7	25
Total		201	146	176	107	472

### Evolution of celery

WGD events occur widely in flowering plants and are one of the most important drivers of genome evolution, the origination of new species, and gene neofunctionalization^54,60^. *Ks* values can be used to estimate the timing of large-scale duplications^61^. The distribution of *Ks* values between celery paralogous pairs displayed two peaks, at 0.025 and 0.385 (Fig. 2b), which indicated recent WGD events in the evolution of celery. Based on the generally accepted evolutionary rate^62^, the WGD events might have occurred at times of ~1.9 and 29.6 Mya.

Using the 28,874 orthologous gene families identified by OrthoMCL from the celery genome and eight other fully sequenced genomes, we constructed a phylogenetic tree to show the relationships among the nine higher plants. The times of divergence among these plant species were also estimated (Fig. 2c). We used the time of divergence between *S. tubersum* and *S.* *lycopersicum* as a calibration point. Celery and carrot diverged from a common ancestor ~35.1 Mya. In addition, we performed a comparative analysis of gene family evolution in the nine plants in the phylogenetic tree. A total of 2131 gene families were expanded in the celery lineage, whereas 2721 gene families had undergone contraction.

### Apigenin biosynthesis pathway in celery

Apigenin is one of the most important flavonoid compounds and exhibits a variety of biological activities and pharmacological effects^3^. Compared with other plants, celery has a higher content of apigenin^6,8^. In light of the flavonoid compounds among the important secondary metabolites of celery, we analyzed the genes involved in the flavonoid biosynthesis pathway. Most of these flavonoid biosynthesis pathway genes were found in our dataset (Supplementary Fig. S6). Here, 41 genes that were putatively involved in the flavonoid biosynthesis pathway, mainly encoding 13 different enzymes, were identified in celery based on the genome sequences (Supplementary Table S10). Compared with the flavonoid biosynthesis genes of *Arabidopsis*, rice, and tomato (Supplementary Tables S10 and S11), the greatest number of genes was found in tomato (49), followed by celery (41), rice (34), and *Arabidopsis* (21). The main flavonoid genes in tomato are the *HCT* and *CCOAOMT* genes, which account for 55% of these genes, whereas the numbers of different genes in other species are relatively diverse.

**Fig. 3:**
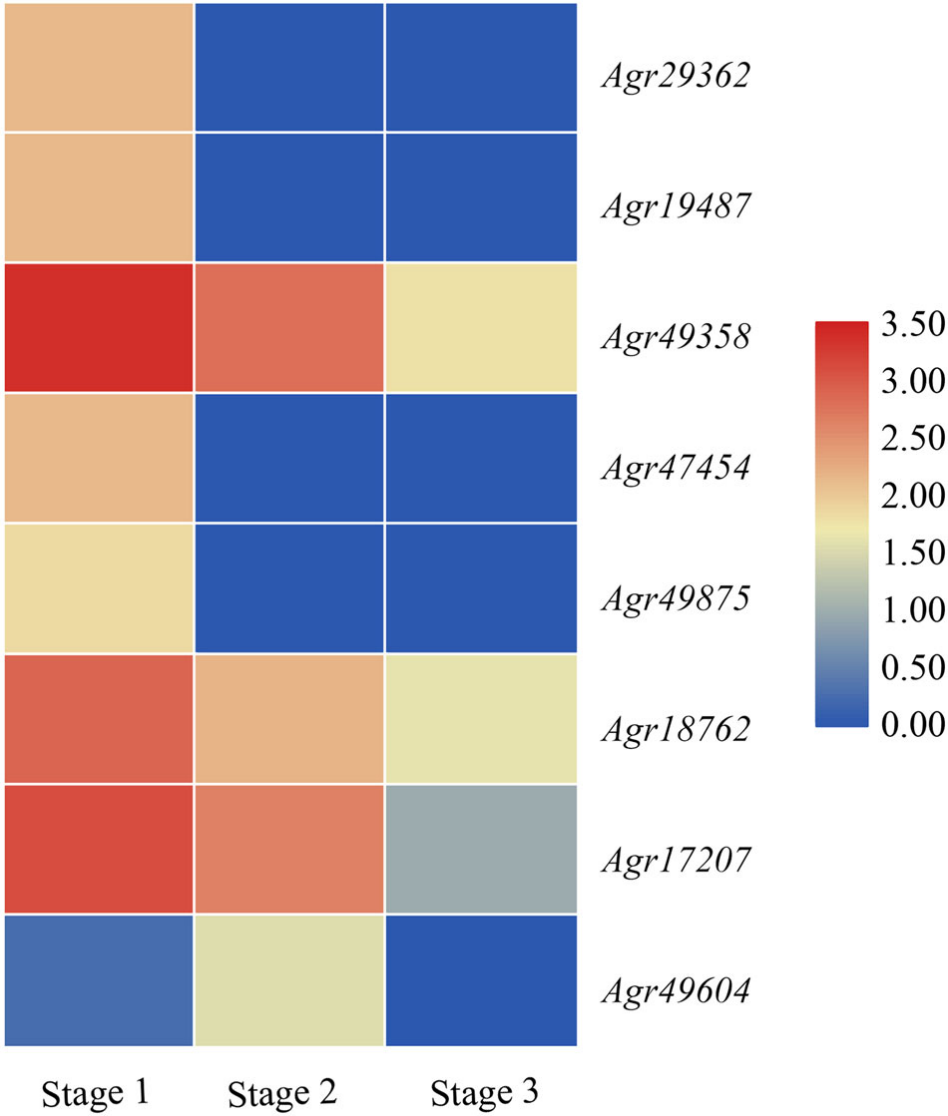
Heatmap of gene transcript abundance in the apigenin pathway at three developmental stages in celery. Stage 1, 35 days after sowing; Stage 2, 50 days after sowing; Stage 3, 60 days after sowing. RPKM values are log2-based. Red and blue indicate high and low expression levels, respectively.

Chalcone synthase (CHS) is a key enzyme in the apigenin biosynthesis pathway, and more genes encoding CHS were found in celery than in other plants. One chalcone isomerase (CHI) was obtained from the celery genome. Flavone synthase I (FNSI) is another enzyme that is necessary for apigenin biosynthesis. Two *FNSI* genes were detected in celery but not in *Arabidopsis*, rice, or tomato. Based on celery RNA-seq data from three developmental stages^63,64^, the expression changes in apigenin-related genes were analyzed, and the results were presented in a heatmap. Most of the apigenin biosynthesis-related genes showed the highest expression levels at early stages, and their expression trends decreased with celery growth and development (Fig. 3). This work extends previous reports on related genes involved in apigenin biosynthesis and provides an overview of apigenin biosynthesis pathways in celery.

### Terpenoid synthase family genes

**Fig. 4:**
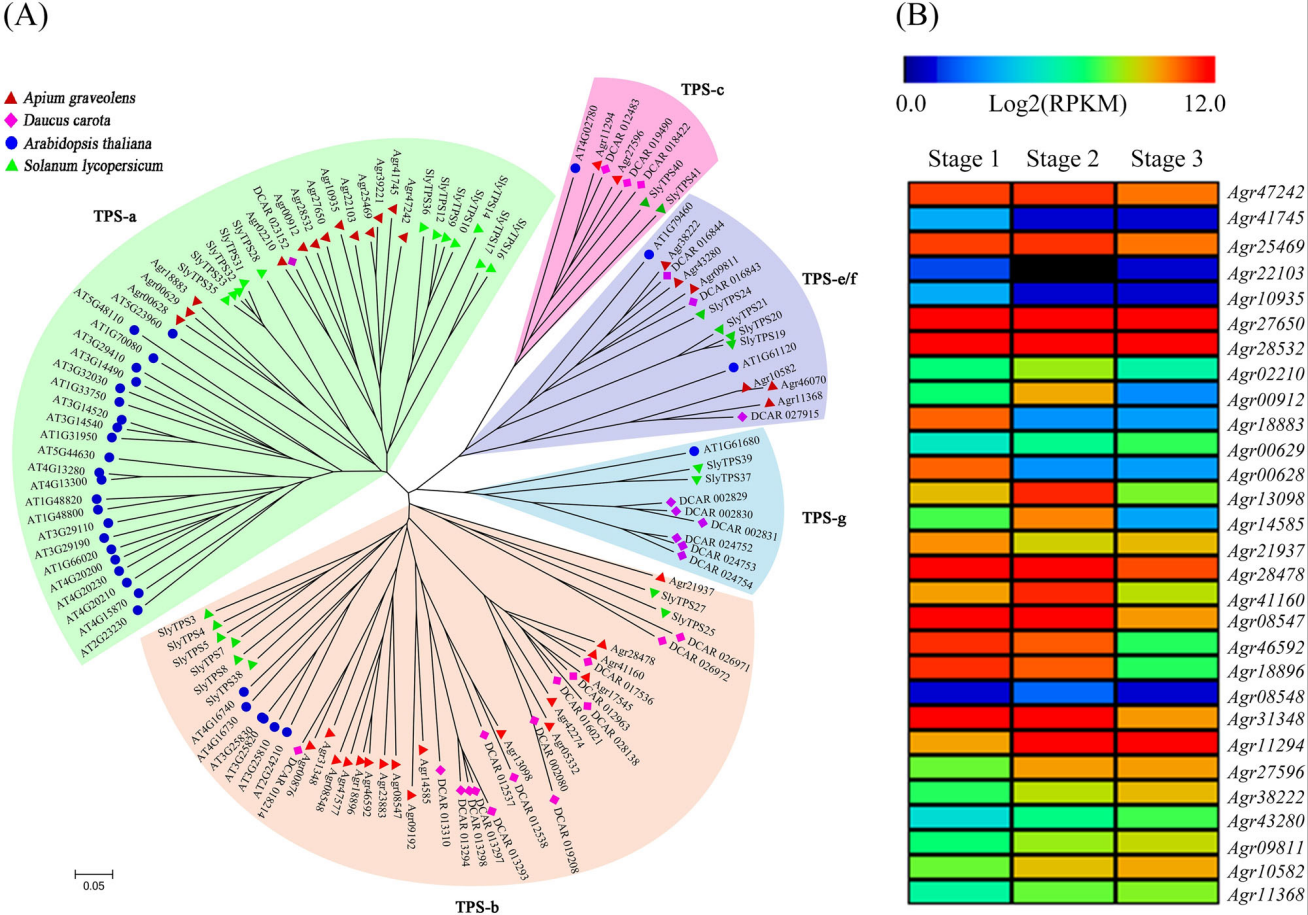
Phylogenetic tree and heatmap of *TPS* proteins in celery. **a** Phylogenetic tree of all TPS proteins among celery, carrot, *Arabidopsis*, and tomato. **b** Heatmap clustering of *TPS* gene transcript abundances at three developmental stages (Stage 1, 35 days after sowing; Stage 2, 50 days after sowing; Stage 3, 60 days after sowing) in celery. The RPKM values are log2-based. Red and black indicate high and low expression levels, respectively.

The flavor of celery is mainly attributed to its terpenoid content. TPSs are the key enzymes that catalyze complex multiple-step cyclization in terpene metabolism. A total of 38 putative TPS proteins were screened in the celery genome by using HMMER software based on the highly conserved domain. To confirm the classification of TPS family proteins in celery, we constructed a phylogenetic tree by aligning the TPS proteins among celery, carrot^20^, *Arabidopsis*^65^, and tomato^66^. All the proteins were divided into five subfamilies: TPS-a, TPS-b, TPS-c, TPS-e/f, and TPS-g (Fig. 4a). In angiosperms, the TPS-b and TPS-a subfamilies account for most of the sesquiterpene synthases and monoterpene synthases, which constitute the majority of TPS proteins. DCAR_023152 (DcTPS1) and DCAR_012963 (DcTPS2) were two TPSs found in carrot belonging to the TPS-a subfamily and TPS-b subfamily, respectively^67^. The TPSs of celery showed the closest phylogenetic relationships with those of carrot, another Apiaceae family plant. In the celery genome, the TPS-b and TPS-a subfamilies contained 17 and 13 members, and only six and two proteins belonged to the TPS-e/f and TPS-c subfamilies, respectively, whereas no proteins clustered within the TPS-g subfamily in celery. Based on previous transcriptome data from celery^63,64^, we further analyzed the expression abundance of *TPS* genes in three developmental stages. As illustrated in Fig. 4b, 30 *TPS* genes were continuously expressed during development. Compared with stage 3, most *TPS* genes exhibited higher transcript abundance in stage 1 and stage 2, suggesting that these genes may be involved in a variety of terpenoid metabolic processes during celery growth and development.

## Discussion

Next-generation sequencing is a powerful method for genome research. With the continuous development of sequencing technology, whole-genome sequencing has been carried out in increasing numbers of species, providing a great deal of biological information for genome mapping, functional gene mining and molecular breeding. Celery is normally classified as a cool-season vegetable and widely consumed around the world, and the cultivation history of celery is longer than 2000 years^2,3^. To understand the genetic system and evolution of Apiaceae, we sequenced the genome of celery, which offers new insights and provides resources for molecular breeding.

Here, we report the first genome data for celery. The genome size was estimated to be 3.18 Gb, which is close to the previous size estimate obtained using flow cytometry of ~3.14 Gb. The genome size of celery is far larger than that of carrot, another Apiaceae species, and is even larger than those of some angiosperm plants, such as *Arabidopsis*, rice and poplar. Celery is one of the sequenced species with the largest genome sizes among those sequenced in recent years, and the celery genome is mainly composed of repetitive sequences, which account for 68.88% of the genome, whereas this percentage is 45.95% in carrot. By comparing the genome components of species with different genome sizes, we found that the proportions of repetitive sequences were obviously higher in species with large genome sizes than in other species (e.g., 80.89% in tea tree^68^ and 76.58% in ginkgo^69^).

WGD events and tandem duplications are the most important determinants of the variation in the genome sizes of angiosperms^54,70^. As two important crops of the Apiaceae family, the evolutionary analysis suggested that celery and carrot may have diverged ~35.1 Mya. In addition, a recent WGD event was deduced to have occurred in celery 1.9 Mya. This genome duplication event not only caused genome expansion in celery, making its genome larger than of carrot, but may also have contributed to the physiological and morphological diversity of the celery lineage.

As a medicinal and edible plant, celery contains various biologically active substances. The biosynthesis and metabolism pathways of these active substances are a major research focus. The genome sequencing data provide new insights into the unique biosynthetic processes of celery, particularly for some important secondary metabolite pathways. Based on the genome sequences, a total of 41 genes that mainly encoded the enzymes in the flavonoid biosynthesis pathway were screened out. Compared with *Arabidopsis*, rice and tomato, the celery genome contains the richest set of apigenin biosynthesis genes, including *CHS*, *F3H*, *F3’H*, and *FNSI*. The duplication and evolution of these genes in celery might be important contributors to enhancing the ability of celery to synthesize flavonoids.

The rich flavor of celery is mainly owing to its high content of terpenoids. In addition, terpenoids play numerous roles in plants, such as functions in resistance to pathogens and pests, acting as the precursors of plant hormones, and participating in plant growth and development^71–73^. The obtained genome sequences helped us to identify *TPS* family genes, which are essential for exploring terpene synthases and terpenoid metabolism. Expression abundance analysis showed that most of the *TPS* genes were strongly expressed in the late developmental stage of celery, which was consistent with the increased expression of *TPS* genes during carrot maturation^67^. Although further research is needed to confirm the regulatory mechanisms of *TPS* genes, the results reveal that *TPS* genes show temporal specificity during different developmental periods. The information and genome sequence resources reported for the celery genome in this study can enhance both fundamental and applied research on celery and other Apiaceae family plants.

## Supplementary information

Supplemental material

## References

[1] Burt S (2004). Essential oils: their antibacterial properties and potential applications in foods - a review. Int. J. Food Microbiol..

[2] Al-Asmari AK, Athar MT, Kadasah SG (2017). An updated phytopharmacological review on medicinal plant of Arab region: *Apium graveolens* Linn. Pharmacogn. Rev..

[3] Li MY (2018). Advances in the research of celery, an important Apiaceae vegetable crop. Crit. Rev. Biotechnol..

[4] Li JW, Ma J, Feng K, Xu ZS, Xiong AS (2019). Transcriptome profiling of β-carotene biosynthesis genes and β-carotene accumulation in leaf blades and petioles of celery cv. Jinnanshiqin. Acta Biochem. Biophys. Sin..

[5] Lin LZ, Lu SM, Harnly JM (2007). Detection and quantification of glycosylated flavonoid malonates in celery, Chinese celery, and celery seed by LC-DAD-ESI/MS. J. Agric. Food Chem..

[6] Hertog MGL, Hollman PCH, Venema DP (1992). Optimization of a quantitative HPLC determination of potentially anticarcinogenic flavonoids in vegetables and fruits. J. Agric. Food Chem..

[7] Feng K (2018). AgMYB2 transcription factor is involved in the regulation of anthocyanin biosynthesis in purple celery (*Apium graveolens* L.). Planta.

[8] Tan GF, Ma J, Zhang XY, Xu ZS, Xiong AS (2017). AgFNS overexpression increase apigenin and decrease anthocyanins in petioles of transgenic celery. Plant Sci..

[9] Funakoshi-Tago M, Nakamura K, Tago K, Mashino T, Kasahara T (2011). Anti-inflammatory activity of structurally related flavonoids, Apigenin, Luteolin and Fisetin. Int. Immunopharmacol..

[10] Huang CS (2013). Protection by chrysin, apigenin, and luteolin against oxidative stress is mediated by the Nrf2-dependent up-regulation of heme oxygenase 1 and glutamate cysteine ligase in rat primary hepatocytes. Arch. Toxicol..

[11] Momin RA, Nair MG (2001). Mosquitocidal, nematicidal, and antifungal compounds from *Apium graveolens* L. seeds. J. Agric. Food Chem..

[12] Tang D, Chen KL, Huang LQ, Li J (2017). Pharmacokinetic properties and drug interactions of apigenin, a natural flavone. Expert Opin. Drug Metab. Toxicol..

[13] Gadermaier G (2011). Molecular characterization of Api g 2, a novel allergenic member of the lipid-transfer protein 1 family from celery stalks. Mol. Nutr. Food Res..

[14] Feng K (2018). Isolation, purification, and characterization of AgUCGalT1, a galactosyltransferase involved in anthocyanin galactosylation in purple celery (*Apium graveolens* L.). Planta.

[15] Kun LI, Zhang DC, Shan You-Xi (2011). The quantitation of flavonoids in leaf and stalk of different celery cultivars and the correlation with antioxidation activity. Acta Horticulturae Sin..

[16] Arabidopsis Genome I (2000). Analysis of the genome sequence of the flowering plant *Arabidopsis thaliana*. Nature.

[17] Velasco R (2010). The genome of the domesticated apple (*Malus* x *domestica* Borkh.). Nat. Genet..

[18] Schnable PS (2009). The B73 maize genome: complexity, diversity, and dynamics. Science.

[19] Huang S (2009). The genome of the cucumber, *Cucumis sativus* L. Nat. Genet..

[20] Iorizzo M (2016). A high-quality carrot genome assembly provides new insights into carotenoid accumulation and asterid genome evolution. Nat. Genet..

[21] Sun DL (2019). Draft genome sequence of cauliflower (*Brassica oleracea* L. var. botrytis) provides new insights into the C genome in Brassica species. Hortic. Res..

[22] Xu ZS, Tan HW, Wang F, Hou XL, Xiong AS (2014). CarrotDB: a genomic and transcriptomic database for carrot. Database.

[23] Rogers SO, Bendich AJ (1985). Extraction of DNA from milligram amounts of fresh, herbarium and mummified plant tissues. Plant Mol. Biol..

[24] Martin Marcel (2011). Cutadapt removes adapter sequences from high-throughput sequencing reads. EMBnet.journal.

[25] Luo R (2012). SOAPdenovo2: an empirically improved memory-efficient short-read de novo assembler. GigaScience.

[26] Simao FA, Waterhouse RM, Ioannidis P, Kriventseva EV, Zdobnov EM (2015). BUSCO: assessing genome assembly and annotation completeness with single-copy orthologs. Bioinformatics.

[27] Stanke M (2006). AUGUSTUS: ab initio prediction of alternative transcripts. Nucleic Acids Res..

[28] Korf I (2004). Gene finding in novel genomes. BMC Bioinformatics.

[29] Xu X (2011). Genome sequence and analysis of the tuber crop potato. Nature.

[30] Slater GS, Birney E (2005). Automated generation of heuristics for biological sequence comparison. BMC Bioinformatics.

[31] Ashburner M (2000). Gene ontology: tool for the unification of biology. The Gene Ontology Consortium. Nat. Genet..

[32] Tatusov RL (2003). The COG database: an updated version includes eukaryotes. BMC Bioinformatics.

[33] Conesa A (2005). Blast2GO: a universal tool for annotation, visualization and analysis in functional genomics research. Bioinformatics.

[34] Ye J (2006). WEGO: a web tool for plotting GO annotations. Nucleic Acids Res..

[35] Lowe TM, Eddy SR (1997). tRNAscan-SE: a program for improved detection of transfer RNA genes in genomic sequence. Nucleic Acids Res..

[36] Nawrocki EP, Kolbe DL, Eddy SR (2009). Infernal 1.0: inference of RNA alignments. Bioinformatics.

[37] Nawrocki EP (2015). Rfam 12.0: updates to the RNA families database. Nucleic Acids Res..

[38] Chen N (2004). Using RepeatMasker to identify repetitive elements in genomic sequences. Curr. Protoc. Bioinformatics.

[39] Li L, Stoeckert CJ, Roos DS (2003). OrthoMCL: identification of ortholog groups for eukaryotic genomes. Genome Res..

[40] Goodstein DM (2012). Phytozome: a comparative platform for green plant genomics. Nucleic Acids Res..

[41] De Bie T, Cristianini N, Demuth JP, Hahn MW (2006). CAFE: a computational tool for the study of gene family evolution. Bioinformatics.

[42] Fu, L. M., Niu, B. F., Zhu, Z. W., Wu, S. T. & Li, W. Z. CD-HIT: accelerated for clustering the next-generation sequencing data. *Bioinformatics* **28**, 3150–3152 (2012). 10.1093/bioinformatics/bts565 PMC3516142 23060610

[43] Edgar RC (2004). MUSCLE: multiple sequence alignment with high accuracy and high throughput. Nucleic Acids Res..

[44] Jones DT, Taylor WR, Thornton JM (1992). The rapid generation of mutation data matrices from protein sequences. Comput. Appl. Biosci..

[45] Kumar S, Stecher G, Tamura K (2016). MEGA7: Molecular evolutionary genetics analysis version 7.0 forbigger datasets. Mol. Biol. Evol..

[46] Wikstrom N, Savolainen V, Chase MW (2001). Evolution of the angiosperms: calibrating the family tree. Proc. Biol. Sci..

[47] Crepet WL, Nixon KC, Gandolfo MA (2004). Fossil evidence and phylogeny: The age of major angiosperm clades based on mesofossil and macrofossil evidence from cretaceous deposits. Am. J. Bot..

[48] Yang Z (1997). PAML: a program package for phylogenetic analysis by maximum likelihood. Comput. Appl. Biosci..

[49] Riano-Pachon DM, Ruzicic S, Dreyer I, Mueller-Roeber B (2007). PlnTFDB: an integrative plant transcription factor database. BMC Bioinformatics.

[50] Eddy SR (2011). Accelerated profile HMM searches. PLoS Comput Biol..

[51] Feng K (2018). CeleryDB: a genomic database for celery. Database.

[52] SanMiguel P (1996). Nested retrotransposons in the intergenic regions of the maize genome. Science.

[53] Vitte C, Panaud O (2005). LTR retrotransposons and flowering plant genome size: emergence of the increase/decrease model. Cytogenet. Genome Res..

[54] Piegu B (2006). Doubling genome size without polyploidization: dynamics of retrotransposition-driven genomic expansions in Oryza australiensis, a wild relative of rice. Genome Res..

[55] DeYoung BJ, Innes RW (2006). Plant NBS-LRR proteins in pathogen sensing and host defense. Nat. Immunol..

[56] Takken FL, Albrecht M, Tameling WI (2006). Resistance proteins: molecular switches of plant defence. Curr. Opin. Plant Biol..

[57] Meyers BC, Kozik A, Griego A, Kuang H, Michelmore RW (2003). Genome-wide analysis of NBS-LRR-encoding genes in *Arabidopsis*. Plant Cell.

[58] Cheng Y (2012). Systematic analysis and comparison of nucleotide-binding site disease resistance genes in maize. FEBS J..

[59] Zhou T (2004). Genome-wide identification of NBS genes in japonica rice reveals significant expansion of divergent non-TIR NBS-LRR genes. Mol. Genet. Genomics.

[60] Van de Peer Y, Maere S, Meyer A (2009). The evolutionary significance of ancient genome duplications. Nat. Rev. Genet..

[61] Blanc G, Wolfe KH (2004). Widespread paleopolyploidy in model plant species inferred from age distributions of duplicate genes. Plant Cell.

[62] Gaut BS, Morton BR, McCaig BC, Clegg MT (1996). Substitution rate comparisons between grasses and palms: Synonymous rate differences at the nuclear gene Adh parallel rate differences at the plastid gene rbcL. Proc. Natl Acad. Sci..

[63] Jia XL (2015). De novo assembly, transcriptome characterization, lignin accumulation, and anatomic characteristics: novel insights into lignin biosynthesis during celery leaf development. Sci. Rep..

[64] Li MY, Wang F, Jiang Q, Ma J, Xiong AS (2014). Identification of SSRs and differentially expressed genes in two cultivars of celery (*Apium graveolens* L.) by deep transcriptome sequencing. Hortic. Res..

[65] Aubourg S, Lecharny A, Bohlmann J (2002). Genomic analysis of the terpenoid synthase (*AtTPS*) gene family of *Arabidopsis thaliana*. Mol. Genet. Genomics.

[66] Falara V (2011). The tomato terpene synthase gene family. Plant Physiol..

[67] Yahyaa M (2015). Identification and characterization of terpene synthases potentially involved in the formation of volatile terpenes in carrot (*Daucus carota* L.) roots. J. Agric. Food Chem..

[68] Xia EH (2017). The tea tree genome provides insights into tea flavor and independent evolution of caffeine biosynthesis. Mol. Plant.

[69] Guan R (2016). Draft genome of the living fossil *Ginkgo biloba*. GigaScience.

[70] El Baidouri M, Panaud O (2013). Comparative genomic paleontology across plant kingdom reveals the dynamics of TE-driven genome evolution. Genome Biol. Evol..

[71] Bohlmann J, Meyer-Gauen G, Croteau R (1998). Plant terpenoid synthases: molecular biology and phylogenetic analysis. Proc. Natl Acad. Sci..

[72] Tholl D (2006). Terpene synthases and the regulation, diversity and biological roles of terpene metabolism. Curr. Opin. Plant Biol..

[73] Yao LX (2019). Proteomic and metabolomic analyses provide insight into the off-flavour of fruits from citrus trees infected with ‘Candidatus Liberibacter asiaticus’. Hortic. Res..

