# Supplementary material for: The genome sequence of celery (Apium graveolens L.), an important leaf vegetable crop rich in apigenin in the Apiaceae family
Source: Hortic Res. 2020 Jan 6;7:9. doi: 10.1038/s41438-019-0235-2 (PMC6944684; doi:10.1038/s41438-019-0235-2)
Supplement: Supplementary file 1 — Supplemental material [file 41438_2019_235_MOESM1_ESM.doc]

**Table S1 Information of the paired-end libraries used in celery sequencing**

| Library insert size | Library number | Clean data (Gb) | Sequence coverage (×) |
| --- | --- | --- | --- |
| 180 bp | 18 | 211.47 | 66.50 |
| 500 bp | 14 | 152.35 | 47.91 |
| 800 bp | 18 | 136.35 | 42.88 |
| 2 kb | 8 | 47.78 | 15.03 |
| 5 kb | 8 | 27.78 | 8.74 |
| 10 kb | 2 | 25.07 | 7.88 |
| Total | 68 | 600.80 | 188.93 |

**Table S2 Estimation of celery genome size based on 17 *k*-mer statistics**

| ***k*-mer** | ***k*-mer Number** | | **Peak Depth** | | **Estimated Genome Size (bp)** | |
| --- | --- | --- | --- | --- | --- | --- |
| 17 | | 410,284,820,271 | | 120 | | 3,419,040,169 |

**Table S3** Quality assessment of the assembled genome of celery using BUSCOs.

| **Type** | **Number** | **Percent (%)** |
| --- | --- | --- |
| Complete BUSCOs (C) | 1,307 | 90.8 |
| Complete and single-copy BUSCOs (S) | 1,195 | 83.0 |
| Complete and duplicated BUSCOs (D) | 102 | 7.8 |
| Fragmented BUSCOs (F) | 37 | 2.6 |
| Missing BUSCOs (M) | 96 | 6.6 |
| Total BUSCO groups searched | 1,440 | 100 |

**Table S4** Statistics of sequenced plant genomes

| Data | *A. graveolens* | *D. carota* | *A. thaliana* | *S. lycopersicum* | *C. sinensis* | *O. sativa* |
| --- | --- | --- | --- | --- | --- | --- |
| Genome size (bp) | 2,372,941,895 | 424,246,664 | 119,481,543 | 781,345,411 | 3,021,230,785 | 374,192,225 |
| Genome GC (%) | 35.35 | 34.80 | 36.06 | 34.05 | 42.31 | 43.57 |
| Exon region size (bp) | 43,970,297 | 38,138,439 | 34,833,833 | 41,982,637 | 42,035,458 | 80,300,819 |
| Exon region (%) | 1.85 | 8.99 | 29.15 | 5.37 | 1.39 | 21.46 |
| Exon region GC (%) | 42.06 | 43.86 | 44.14 | 41.10 | 44.55 | 52.89 |
| Gene number | 34,277 | 32,113 | 27,416 | 34,727 | 36,951 | 57,939 |
| mRNA number | 34,277 | 32,113 | 35,386 | 34,727 | 36,951 | 68,563 |
| Average mRNA length (bp) | 3,267 | 3,366 | 1,230 | 1,515 | 3,549 | 1323 |
| Exon number | 180,591 | 160,786 | 147,494 | 160,007 | 177,365 | 246,667 |
| Average exon length (bp) | 243.48 | 237.20 | 236.17 | 262.38 | 237 | 325.54 |
| Exon No. per gene | 5.27 | 5.01 | 5.38 | 4.61 | 4.80 | 4.26 |
| Gene No. per 100kb | 1.44 | 7.6 | 22.95 | 4.44 | 1.22 | 15.48 |

**Table S5 Summary of repeat elements identified in *A. graveolens*, *C. sinensis*, *G. bilob*a, *D. carota* and *J. curcas* genomes**

| Types | *A. graveolens* | *C. sinensis* | *G. biloba* | *D. carota* | *J. curcas* |
| --- | --- | --- | --- | --- | --- |
| Genome size | 2.21 Gb | 3.02 Gb | 10.61 Gb | 421.50 Mb | 320.50 Mb |
| Percentage for repeat elements to genome | 68.88 % | 80.89 % | 76.58 % | 45.95 % | 49.81 % |
| DNA TE | 3.30 % | 7.26 % | 3.34 % | 13.60 % | 4.08 % |
| LTR | 44.07 % | 55.09 % | 60.65 % | 27.43 % | 31.98 % |
| Other repeats | 21.51 % | 18.54 % | 12.59 % | 4.92 % | 13.75 % |

**Table S6 Number of gene models with homology or functional classification in celery genome**

| Annotation Database | Sequence number | Annotation number |
| --- | --- | --- |
| Nr | 34,135 | 39,798 |
| InterPro | 25,712 | 39,311 |
| GO | 16,920 | 38,141 |
| KEGG | 9,463 | 9,463 |
| All database annotation | 34,143 | - |

**Table S7 Gene family clustering**

Genes from celery genome were collected and aligned to each other using BLASTP.

| Species | Genes number | Genes in families | Unclustered genes | Family number | Average genes per family |
| --- | --- | --- | --- | --- | --- |
| Celery | 34,277 | 27,549 | 6,728 | 15,164 | 1.82 |

**Table S8 Significantly overrepresented Gene Ontology terms in gene families**

Just list the top 10 gene families.

| Cluster ID | Term ID | Description | P value | Adjust p value | Count |
| --- | --- | --- | --- | --- | --- |
| Group 4 | GO:0008037 | cell recognition | 1.776881e-175 | 1.747266e-174 | 84 |
| Group 5 | GO:0004252 | serine-type endopeptidase activity | 1.861999e-117 | 2.979199e-116 | 52 |
| Group 17 | GO:0050660 | flavin adenine dinucleotide binding | 2.338396e-98 | 3.975273e-97 | 50 |
| Group 8 | GO:0006468 | protein phosphorylation | 9.086559e-30 | 3.998086e-28 | 38 |
| Group 12 | GO:0008270 | zinc ion binding | 4.183203e-44 | 3.346563e-43 | 37 |
| Group 6 | GO:0005515 | protein binding | 9.241051e-26 | 3.973652e-24 | 37 |
| Group 64 | GO:0043531 | ADP binding | 5.108546e-65 | 9.195383e-64 | 35 |
| Group 20 | GO:0016887 | ATPase activity | 5.341004e-60 | 1.495481e-58 | 32 |
| Group 69 | GO:0030599 | pectinesterase activity | 6.378456e-70 | 7.335224e-69 | 32 |
| Group 19 | GO:0008270 | zinc ion binding | 5.245331e-37 | 3.671732e-36 | 31 |

**Table S9** Distribution of GO term classification of celery-specific genes. The top 20 terms were represented.

| **Category** | **GO term** | **Description** | **Number of genes** |
| --- | --- | --- | --- |
| Molecular Function | GO:0005515 | protein binding | 198 |
| Molecular Function | GO:0008270 | zinc ion binding | 135 |
| Molecular Function | GO:0003677 | DNA binding | 71 |
| Molecular Function | GO:0004672 | Protein kinase activity | 57 |
| Molecular Function | GO:0005524 | ATP binding | 51 |
| Molecular Function | GO:0003676 | nucleic acid binding | 35 |
| Molecular Function | GO:0046983 | protein dimerization activity | 24 |
| Molecular Function | GO:0008234 | cysteine-type peptidase activity | 22 |
| Molecular Function | GO:0003678 | DNA helicase activity | 18 |
| Molecular Function | GO:0003723 | RNA binding | 17 |
| Cellular Component | GO:0005634 | nucleus | 20 |
| Cellular Component | GO:0016020 | membrane | 17 |
| Biological Process | GO:0006468 | protein phosphorylation | 57 |
| Biological Process | GO:0055114 | oxidation-reduction process | 48 |
| Biological Process | GO:0006355 | regulation of transcription, DNA-templated | 40 |
| Biological Process | GO:0006508 | proteolysis | 35 |
| Biological Process | GO:0006457 | protein folding | 19 |
| Biological Process | GO:0006281 | DNA repair | 18 |
| Biological Process | GO:0000723 | telomere maintenance | 18 |
| Biological Process | GO:0000413 | protein peptidyl-prolyl isomerization | 17 |

**Table S10** Number of predicted genes encoding enzymes of flavonoid biosysthesis in celery, *Arabidopsis*, tomato, and rice

| Species | CHS | CHI | F3H | FNSI | FLS | CYP73A | PKR | CYP75A | LDOX | ANR |
| --- | --- | --- | --- | --- | --- | --- | --- | --- | --- | --- |
| *A. graveolens* | 8 | 1 | 5 | 2 | 3 | 2 | 0 | 1 | 1 | 0 |
| *A. thaliana* | 1 | 2 | 1 | 0 | 5 | 1 | 0 | 0 | 2 | 1 |
| *O. sativa* | 2 | 1 | 1 | 0 | 1 | 3 | 0 | 1 | 2 | 7 |
| *S. lycopersicum* | 5 | 2 | 1 | 0 | 1 | 3 | 0 | 1 | 1 | 1 |
| Species | DICGT | AS1 | C12RT1 | LAR | HCT | CYP98A | DFR | CCOAOMT | F3’H |  |
| *A. graveolens* | 0 | 0 | 0 | 0 | 4 | 2 | 2 | 7 | 3 |  |
| *A. thaliana* | 0 | 0 | 0 | 0 | 1 | 1 | 1 | 4 | 1 |  |
| *O. sativa* | 0 | 0 | 0 | 1 | 8 | 2 | 1 | 2 | 2 |  |
| *S. lycopersicum* | 0 | 0 | 0 | 0 | 15 | 5 | 1 | 12 | 1 |  |

CHS, chalcone synthase; CHI, chalcone isomerase; F3H, Flavanone-3-hydroxylase;

FNSI, flavone synthase I; FLS, Flavonolsynthase/flavanone3-hydroxylase;

CYP73A, trans-cinnamate4-monooxygenase; PKR, Polyketide reductase;

CCOAOMT, caffeoyl-CoAO-methyltransferase; CYP75A, flavonoid3', 5'-hydroxylase;

LDOX, leucoanthocyanidin dioxygenase; ANR, anthocyanidin reductase;

DICGT, chalcononaringenin2'-O-glucosyltransferas; ASI, aureusidin synthase;

C12RT1, flavanone7-O-glucoside2''-O-beta-L-rhamnosyltransferase;

LAR, leucoanthocyanidin reductase; HCT, shikimateO- hydroxycinnamoyltransferase;

CYP98A, coumaroylquinate (coumaroylshikimate)3'-monooxygenase;

DFR, dihydroflavonol4-reductase/ flavanone4-reductase; F3’H, flavonoid3'-monooxygenase.

**Table S11 Predicted genes encoding enzymes of flavonoid biosynthesis in celery, *Arabidopsis*, rice, and tomato**

| Genus | Gene | *CHS* | *CHI* | *F3H* | *FNSI* | *FLS* | *CYP73A* | *PKR* | *F3’H* | *LDOX* | *ANR* |
| --- | --- | --- | --- | --- | --- | --- | --- | --- | --- | --- | --- |
|  | EC | 2.3.1.74 | 5.5.1.6 | 1.14.11.9 | 1.14.11.22 | 1.14.11.23 | 1.14.13.11 | 2.3.1.70 | 1.14.13.21 | 1.14.11.19 | 1.3.1.77 |
| *A. thaliana* | | AT5G13930 | AT5G66220  AT3G55120 | AT3G51240 |  | AT5G08640  AT5G63590  AT5G43935  AT5G63600  AT5G63595 | AT2G30490 |  | AT5G07990 | AT4G22870  AT4G22880 | AT1G61720 |
| *A. graveolens* | | Agr00142  Agr36670  Agr29362  Agr19487  Agr49358  Agr47454  Agr49875  Agr44334 | Agr18762 | Agr31087  Agr08162  Agr35080  Agr45928  Agr39987 | Agr17207  Agr49604 | Agr19063  Agr34935  Agr29706 | Agr38795  Agr28893 |  | Agr25333  Agr06001  Agr06101 | Agr31287 |  |
| *S. lycopersicum* | | 101245061  101263032  101266107  778295, 778294 | 101249265  101250693 | 100736482 |  | 101249699 | 101262919  101244196  101244496 |  | 101266618 | 101251607 | 101263493 |
| *O. sativa* | | Os11t0530600  Os07t0214900 | Os03t0819600 | Os04t0662600 |  | Os02t0767300 | Os05t0320700  Os02t0467600  Os02t0467000 |  | Os10t0320100  Os10t0317900 | Os01t0372500  Os06t0626700 | Os04t0630800  Os04t0631000  Os04t0630300  Os04t0630400  Os04t0630600  Os04t0630900  Os04t0630100 |

**Continue:**

| Genus | Gene | DICGT | AS1 | C12RT1 | LAR | HCT | CYP98A | DFR | CCOAOMT | CYP75A |
| --- | --- | --- | --- | --- | --- | --- | --- | --- | --- | --- |
|  | EC | 2.4.1.- | 1.21.3.6 | 2.4.1.236 | 1.17.1.3 | 2.3.1.133 | 1.14.13.36 | 1.1.1.219 | 2.1.1.104 | 1.14.13.88 |
| *A. thaliana* | |  |  |  |  | AT5G48930 | AT2G40890 | AT5G42800 | AT4G34050  AT1G24735  AT1G67980  AT4G26220 |  |
| *A. graveolens* | |  |  |  |  | Agr43589, Agr06463, Agr03965  Agr20312 | Agr00692, Agr13350 | Agr24429  Agr17988 | Agr47053,Agr15807  Agr24121,Agr22019  Agr43197,Agr22021  Agr06777 | Agr25333 |
| *S. lycopersicum* | |  |  |  |  | 544249, 101256271, 101255552  101255851, 101256149, 101249522  101253503, 101266953, 101248691 101245674, 101244961, 101249389  101253556, 101253859, 101267379 | 101246092, 01261765  101262063,101262367  101247849 | 544150 | 101252173, 101265690  101253032, 101255734  101260278, 101266196  100134898, 101245167  101248393, 101248678  101265187, 101265977 | 100736504 |
| *O. sativa* | |  |  |  | Os03t0259400 | Os02t0611800, Os04t0500700  Os08t0205000, Os06t0184900  Os06t0185500, Os05t0116800  Os08t0543400, Os09t0422000 | Os05t0494000  Os10t0196100 | Os01t0633500 | Os09t0481400  Os06t0165800 | Os03t0367101 |


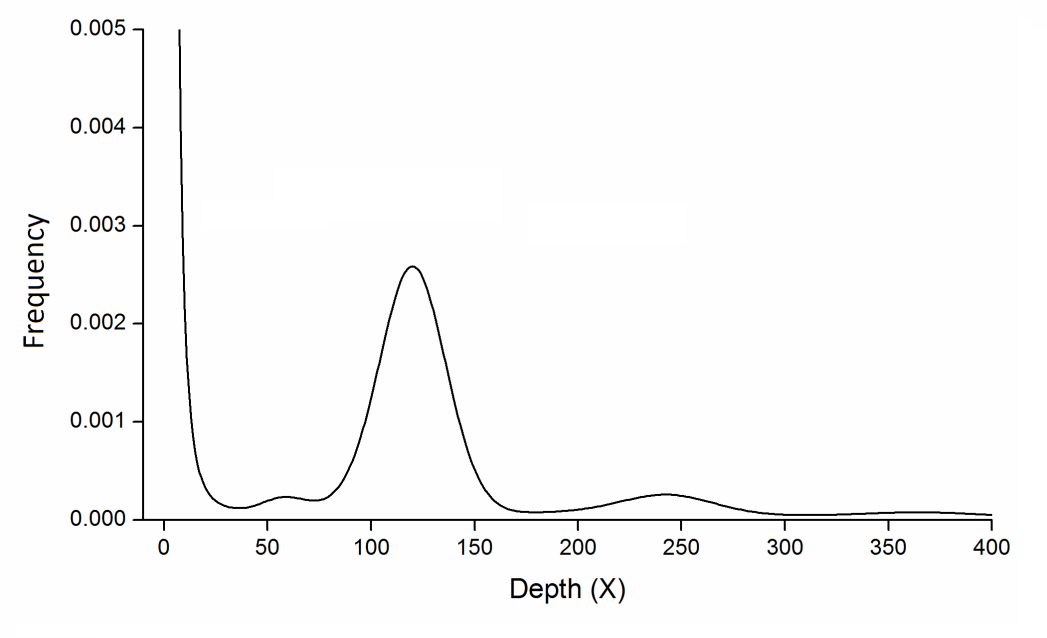


**Fig. S1 Distribution curve of 17 *K*-mer depth frequency**

The genome size was estimated as (total *K*-mer number)/(the volume peak).

**
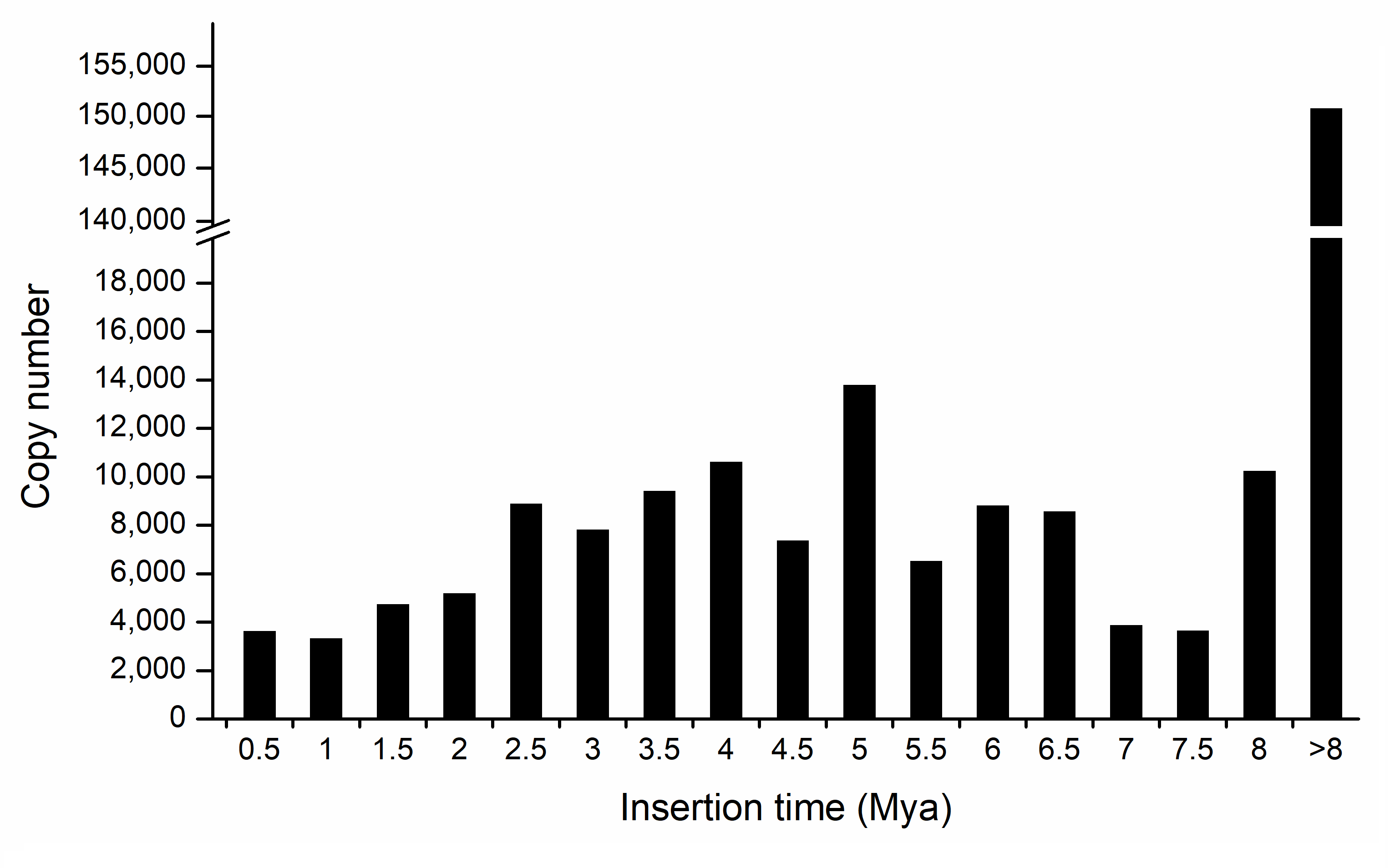
**

**Fig. S2** The LTR insertion time in celery genome.


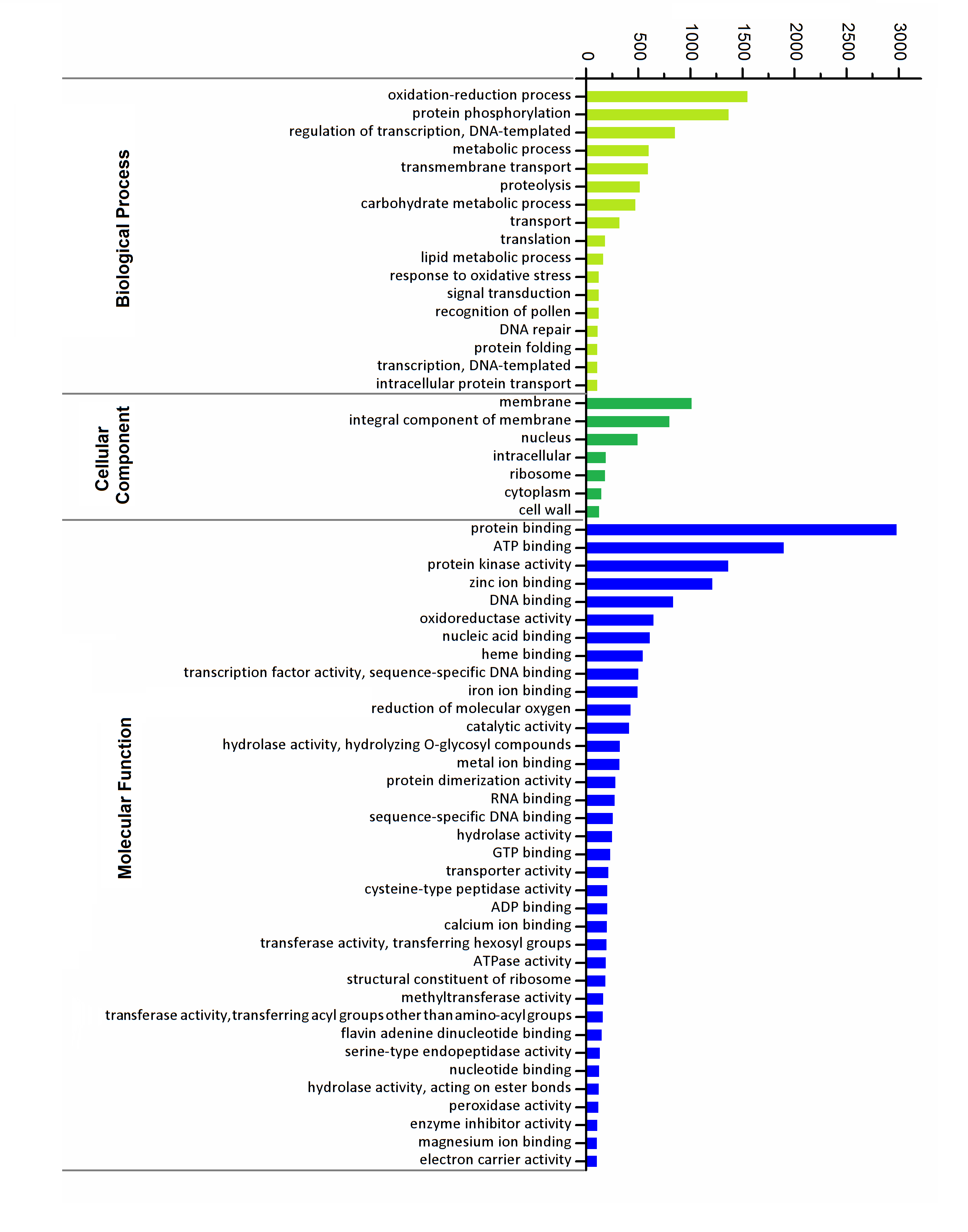


**Fig. S3** Distribution of celery genes among GO function classes

Just list the GO terms contain more than 100 genes.


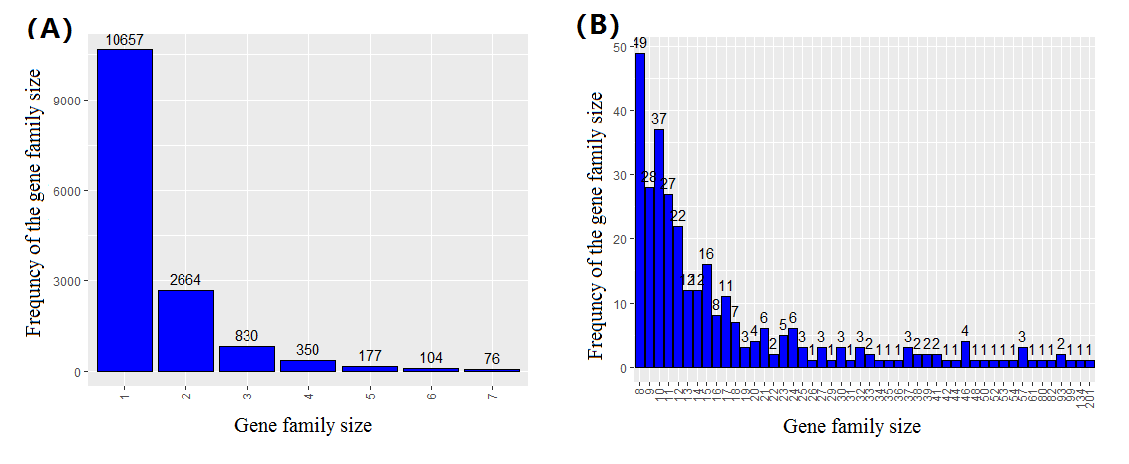


**Fig. S4 Distribution of frequency of gene family size in celery genome**

(A) The gene family size range 1~7.

(B) The gene family size over 7.


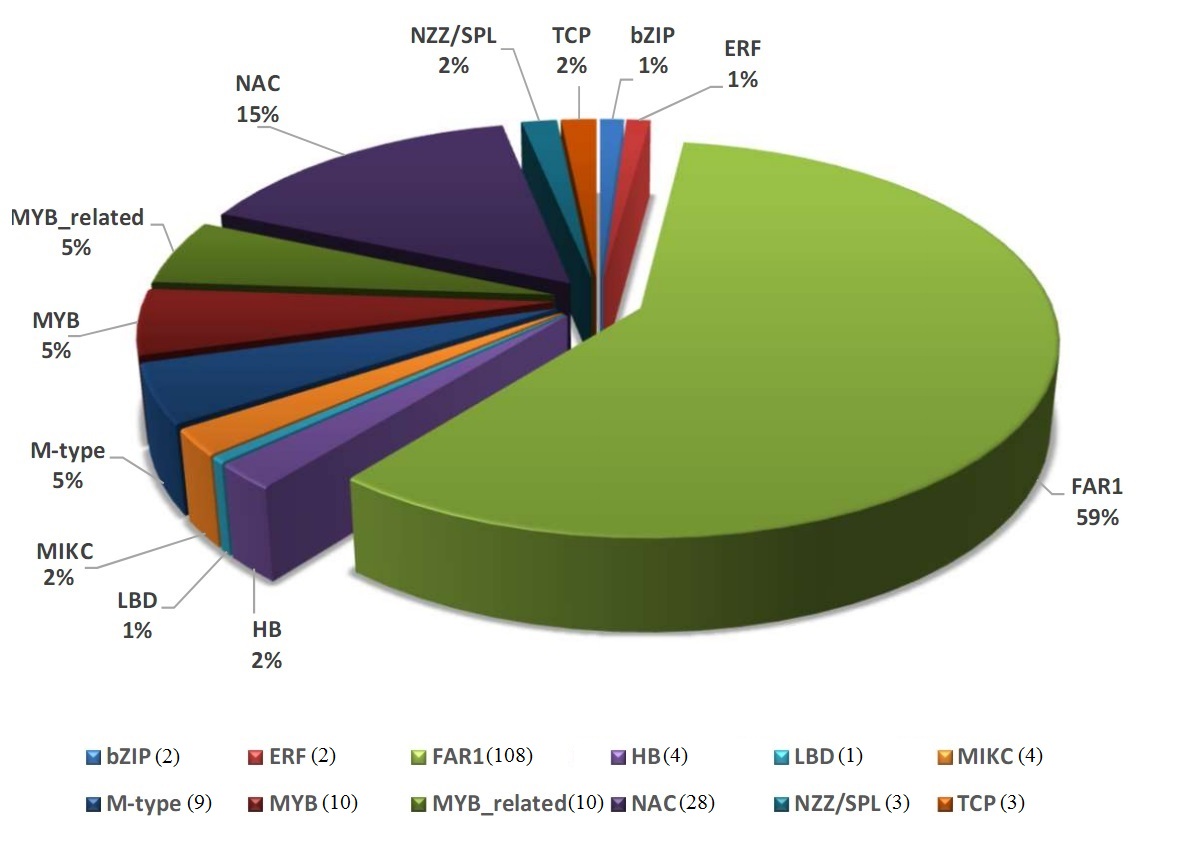


**Fig. S5** Distribution of celery-specific genes among transcription factor families


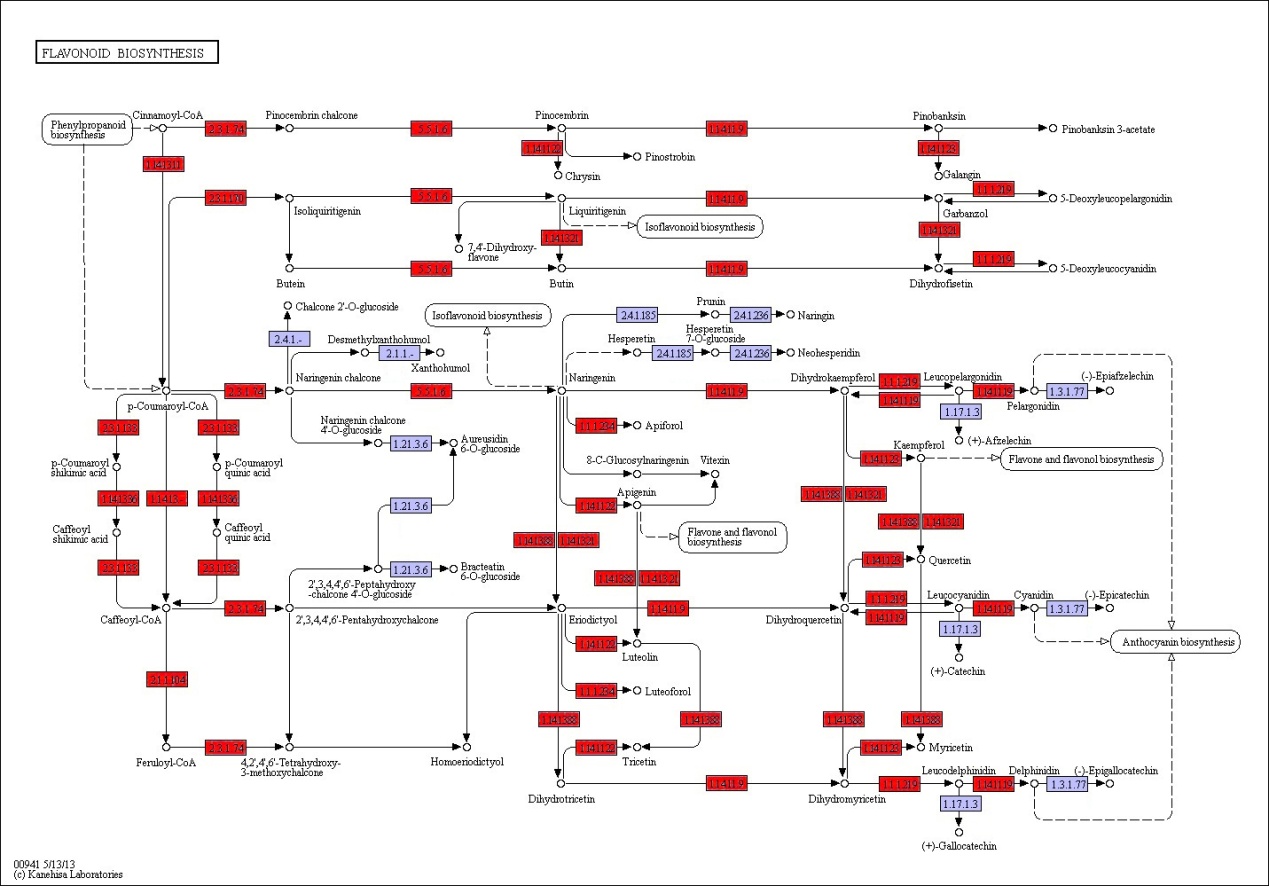


**Fig. S6 The KEGG pathway of flavonoid biosynthesis in celery**

The red box represents that the genes encoding these enzymes are predicted in celery genome.
